# Comparing Laparoscopic Total Extraperitoneal and Lichtenstein Mesh Repair for Inguinal Hernias: A Focus on Patient Outcomes

**DOI:** 10.7759/cureus.57373

**Published:** 2024-04-01

**Authors:** Amar Varshney, Vipin Kawatra, Unnati Watal, Nevin Thyparambil Abraham, Manisha Avinash, Arun Shreenivas Pugalendhi, Shrey Rana

**Affiliations:** 1 Department of Surgery, 7 Air Force Hospital, Kanpur, IND; 2 Department of Surgery, Indian Naval Hospital Ship (INHS) Sanjeevani, Kochi, IND; 3 Department of Surgery, Terna Medical College, Mumbai, IND; 4 Department of Surgery, Dr. Somervell Memorial Medical College, Karakonam, IND; 5 Department of Surgery, The Oxford Medical College Hospital and Research Center, Attibele, IND; 6 Department of Surgery, Government Medical College and Employee State Insurance (ESI) Hospital, Coimbatore, IND; 7 Department of Surgery, Tver State Medical University, Tver, RUS

**Keywords:** hernia repair techniques, postoperative pain, inguinal hernia, laparoscopic total extraperitoneal, lichtenstein tension-free mesh hernioplasty

## Abstract

Background: This study aimed to evaluate post-operative outcomes by comparing factors such as post-operative pain, duration of hospitalization, time needed to resume normal and full activities, and complications between laparoscopic total extraperitoneal (TEP) and Lichtenstein tension-free mesh hernioplasty or repair (LMR) for inguinal hernias.

Materials and methods: A prospective study was conducted involving male patients undergoing either LMR or laparoscopic TEP mesh repair, with 30 patients in each group. The study assessed post-operative pain, duration of hospital stay, return to normal activities, and complications. Pain scores were monitored at regular intervals using a visual scale. Before discharge, patients' ability to perform self-care activities was evaluated using the Katz index of independence in activities of daily living. Outpatient follow-up was conducted on day 14, one month, three months, and six months post-surgery.

Results: Post-operative pain scores were significantly higher among LMR patients compared to TEP patients until the 14th day post-surgery (p < 0.001). However, pain levels became comparable after that. There were no notable differences in pain scores between unilateral and bilateral hernias. TEP patients experienced significantly shorter hospital stays (p < 0.001) and quicker resumptions of self-care (p < 0.001), light work (p < 0.02), and full work (p < 0.03) compared to LMR patients.

Conclusion: Laparoscopic TEP repair offers advantages over Lichtenstein mesh repair in terms of reduced postoperative pain, shorter hospital stays, and faster recovery to normal activities. These findings can guide clinicians and patients in making informed decisions regarding hernia repair techniques.

## Introduction

Inguinal hernia repair ranks among the most prevalent surgical procedures conducted worldwide, with an estimated 20 million surgeries performed annually [1]. Two notable techniques that have gained prominence among the array of options are laparoscopic total extraperitoneal (TEP) repair and Lichtenstein tension-free mesh hernioplasty or repair (LMR) for inguinal hernia treatment [2]. The choice between these techniques often depends on factors such as surgeon preference, patient characteristics, and institutional practices. While both methods aim to achieve durable hernia repair and minimize postoperative complications, there is ongoing debate regarding their comparative effectiveness in terms of patient outcomes.

Numerous studies have investigated the outcomes of laparoscopic TEP and Lichtenstein mesh repair, but there remains a need for comprehensive analyses that directly compare the outcomes of these approaches [3-6]. This study seeks to enhance the current body of knowledge by conducting a comprehensive comparison of laparoscopic TEP and Lichtenstein mesh repair techniques for inguinal hernias, with a specific emphasis on patient outcomes. Recognizing the importance of informed decision-making for both clinicians and patients and understanding the relative advantages and disadvantages of each approach are essential.

This study seeks to compare post-operative outcomes such as pain levels, length of hospital stay, time taken to resume normal and full activity, as well as complications between laparoscopic TEP and LMR for inguinal hernias. The aim is to determine the most effective treatment modality by evaluating the morbidity associated with these procedures. Understanding the level and duration of pain experienced by patients after surgery can significantly influence their recovery experience [7]. The length of hospitalization can impact costs and recovery time. Comparing the typical hospital stay for both techniques provides valuable information for patients and healthcare providers. The time it takes for patients to resume their regular activities after surgery is a crucial indicator of recovery progress [8-10].

## Materials and methods

This prospective study was conducted at the surgery department of 7 Air Force Hospital, Kanpur, over two years, from June 2020 to June 2022. Ethical clearance was obtained from the Institutional Ethics Committee before commencing the research (IEC/546/2019). The study included 30 male patients in each group admitted for mesh hernioplasty procedures, either Lichtenstein or laparoscopic TEP mesh repair under general anaesthesia. Patients who were excluded from the study were those with recurrent or complicated hernias, congenital hernias, individuals classified as American Society of Anesthesiologists (ASA) grade III and above, and those with a prior history of failed laparoscopic hernia repair.

Patients meeting the inclusion criteria were grouped into two categories: group A comprised patients who underwent Lichtenstein mesh hernioplasty (LMR). At the same time, group B consisted of those who underwent laparoscopic TEP mesh repair. Post-operatively, patients were monitored daily for pain scores and complications until discharge. All surgeries were performed by a single surgeon who had a minimum experience of 50 similar procedures. A standard-size prolene mesh measuring 15 cm × 7 cm for open repairs and 15 cm × 15 cm for laparoscopic procedures was used. Post-operative pain was evaluated using the Visual Analogue Scale (VAS) on the day of surgery and subsequently every six hours [11]. Analgesics were administered based on the patient's needs.

Surgical complications, such as wound haematoma, infection, vascular injuries (including femoral vessels, testicular artery, and pampiniform venous plexus), vas deferens injury, ischemic orchitis, and testicular atrophy, were thoroughly documented. Before discharge, patients' ability to perform self-care activities was evaluated using the Katz index of independence in activities of daily living [12]. Discharge criteria comprised achieving a score of 6 on the Katz index, indicating independence, a pain score below 5 without requiring injectable analgesia, and a well-healed surgical wound free from signs of infection. Follow-up appointments were arranged for outpatients on day 14, one month, three months, and six months following surgery to monitor pain levels and post-discharge complications, which included surgical site infection, seroma/haematoma formation, and recurrence. The assessment of surgical site infections was done using the Southampton scoring system.

The data were presented as means and percentages. Statistical analysis was performed using SPSS version 24.0 software, including the chi-square test, independent sample t-test, and Mann-Whitney U test.

## Results

Table 1 shows the baseline characteristics of the study population, divided into groups A (LMR) and B (TEP).

**Table 1:** Baseline characteristics of the study population

Variables	Group A (LMR), n=30 (%)	Group B (TEP), n=30 (%)
Mean age (years)	55.7 ± 16.01	48.93 ± 15.49
Age range (years)	27–75	21–77
Diagnosis		
Right inguinal hernia	16 (53.33 %)	12 (40 %)
Left inguinal hernia	14 (46.66%)	8 (26.66%)
Bilateral inguinal hernia	0 (0.00%)	10 (33.33%)

In group A (LMR), which consisted of 30 male participants, the average age was 55.7 years with a standard deviation of 16.01 years. The age range varied from 27 to 75 years. In terms of diagnosis distribution, 16 participants (53.33%) had a right-sided inguinal hernia, 14 (46.66%) had a left inguinal hernia, and none had bilateral inguinal hernias.

In group B (TEP), which comprised 30 male participants, the average age was 48.93 years with a standard deviation of 15.49 years. The age ranged from 21 to 77 years. As for the diagnosis distribution, 12 participants (40%) had a right inguinal hernia, 8 (26.66%) had a left inguinal hernia, and 10 (33.33%) had bilateral inguinal hernias.

Table 2 presents a comparison of post-operative pain levels between laparoscopic total extraperitoneal (group B) and Lichtenstein mesh repair (group A) for inguinal hernia at different time intervals. Pain intensity is depicted by the median VAS scores.

**Table 2:** Comparison of the post-operative pain in laparoscopic total extraperitoneal and Lichtenstein mesh repair for inguinal hernia *P value<0.05 is statistically significant (Mann-Whitney U test).

Day post-surgery	Time (hours)	Median VAS		P-value
		Group A (LMR)	Group B (TEP)	
Day 0	18:00	7.5	6	<0.001*
	24:00	6.5	5	<0.001*
Day 1	18:00	5	4	<0.001*
	24:00	5	4	<0.001*
Day 2	18:00	5	4	0.206
	24:00	3	4	0.335
Day 3	18:00	4	4.5	0.999
	24:00	3	4	0.549
14th day		3	2	0.253
1st month		2.5	2	0.131
3rd month		1	1	0.843
6th month		0	0	0.957

On day 0, the median VAS score for group A was 7.5, which was notably higher than the score of 6 observed in group B (p < 0.001*). By day 1, group A showed a median VAS score of 5, whereas group B had a score of 4 (p < 0.001*). Subsequently, pain levels gradually decreased in both groups, although group A consistently exhibited significantly higher levels compared to group B until the 14th day. However, starting from the 14th day onward, there was no statistically significant disparity in pain levels between the two groups, as evidenced by the p-values.

Table 3 shows the post-operative median pain scores for unilateral (group UL) and bilateral (group BL) hernias at different time points post-surgery, measured using the VAS. On day 0, there was no notable distinction in median VAS scores between group UL and group BL, with scores of 6 and 7, respectively (p = 0.281). Similarly, on subsequent days 1 and 2, there were no statistically significant variations in pain scores between the two groups. In summary, the findings indicate that there were no significant discrepancies in post-operative pain scores between unilateral and bilateral hernias across the measured time points following surgery.

**Table 3:** Post-operative median pain scores in unilateral and bilateral hernias P-value was found using Mann-Whitney U test.

Day post-surgery	Time (hours)	Median VAS		P-value
		Group UL	Group BL	
Day 0	18:00	6	7	0.281
	24:00	5	6	0.171
Day 1	18:00	4	4	0.754
	24:00	4	4	0.510
Day 2	18:00	4	4.5	0.823
	24:00	4	4	1.00
Day 3	18:00	4	4	0.857
	24:00	4	4	0.666
14th day		2	2.5	0.361
1st month		2	2	0.979
3rd month		1	1	0.684
6th month		0	0.5	0.248

Table 4 compares post-operative outcomes between laparoscopic TEP and LMR for inguinal hernia, focusing on various parameters.

**Table 4:** Comparison of the post-operative outcome in laparoscopic total extraperitoneal and Lichtenstein mesh repair for inguinal hernia *P-value < 0.05 is statistically significant (unpaired T-test).

Variables	Group A (LMR), n=30 (%)	Group B (TEP), n=30 (%)	P-value
Mean hospital stay in days	4.53 ± 1.04	3.30 ± 0.59	0.001*
Mean days required to return to self-care activities	3.73±0.9	2.7±1.12	0.001*
Mean days required to return to light work	16.2±2.21	14.43±3.46	0.023*
Mean days required to return to full work	86.33±8.5	80.71±10.51	0.03*

The mean duration of hospital stay was significantly shorter in group B compared to group A, with values of 3.30 ± 0.59 days and 4.53 ± 1.04 days, respectively (p < 0.001*). Regarding the mean duration required to return to self-care activities, group B also exhibited a significant advantage over group A, with 2.7 ± 1.12 days compared to 3.73 ± 0.9 days (p < 0.001*). In terms of returning to light work, the mean duration required was notably lower in group B (TEP) compared to group A (LMR), with values of 14.43 ± 3.46 days and 16.2 ± 2.21 days, respectively (p = 0.023*). Similarly, for returning to full work, group B (TEP) demonstrated a quicker recovery compared to group A (LMR), with mean durations required of 80.71 ± 10.51 days and 86.33 ± 8.5 days, respectively (p = 0.03*).

## Discussion

The study findings provide valuable insights into the comparative effectiveness of different surgical approaches for inguinal hernia repair. These results are instrumental in guiding clinical decision-making and improving patient outcomes.

Our study indicates that laparoscopic TEP repair has diminished post-operative pain levels compared to LMR, especially in the immediate period after surgery. This finding is consistent with prior studies suggesting that laparoscopic approaches offer benefits such as decreased post-operative pain and accelerated recovery [13]. The reduced pain experienced following TEP repair could contribute to enhanced patient satisfaction and a swifter resumption of normal activities. Two meta-analyses conducted by Koning et al. and Bobo et al. comparing postoperative pain levels in laparoscopic TEP repair and LMR also corroborate these findings [14,15].

This current study provides further insight into the post-operative pain experience, specifically comparing unilateral and bilateral hernias. Interestingly, there were no significant differences in pain scores between unilateral and bilateral hernias at various time points post-surgery. This finding suggests that the surgical approach may have a more significant impact on post-operative pain than the type of hernia (unilateral or bilateral). However, further research is warranted to explore this relationship in more detail.

The study also underscores various significant post-operative outcomes, such as hospital stay and the duration to resume self-care activities, light work, and full work. The findings distinctly favour laparoscopic TEP repair over LMR, showing shorter hospital stays and quicker recovery times, similar to previous research [16]. Studies conducted by Rathod et al. and Langeveld et al. similarly support these conclusions within the TEP group [17,18]. Patients undergoing TEP repair experienced significantly shorter hospital stays and returned to self-care activities, light work, and full work sooner compared to those undergoing LMR. Studies have shown that TEP repairs can effectively decrease postoperative pain, shorten hospital stays, and expedite patient recovery [19-22].

These findings underscore the clinical benefits of laparoscopic techniques, which may lead to reduced healthcare costs, improved patient satisfaction, and enhanced overall recovery outcomes. Overall, the findings from these tables support the growing body of evidence favouring laparoscopic approaches for inguinal hernia repair. However, it is essential to consider individual patient factors, surgeon expertise, and institutional resources when selecting the most appropriate surgical approach.

The limitations of the study include its reliance on data from a single centre, which may not be representative of broader populations or settings, and TEP requires a longer learning curve. Extensive follow-up is required to study the impact of the prosthetic materials used in laparoscopic repair. Additionally, the small sample size could limit the generalizability of the findings.

## Conclusions

In conclusion, the study results offer strong evidence favouring laparoscopic TEP repair over LMR for managing inguinal hernias. Analysis of post-operative outcomes highlighted several notable benefits linked with the laparoscopic method, such as decreased post-operative pain, shorter hospital stays, and accelerated recovery periods.
